# Colorectal cancer models with increased metastasis

**Published:** 2014-03

Most deaths from colorectal cancer (CRC) are associated with metastatic disease. However, visible metastases rarely form in mouse models of CRC that are generated by injection of human CRC cell lines into the caecum (orthotopic injection). Here, Alamo et al. show that subcutaneous preconditioning increases invasion and metastatic dissemination in mouse CRC models. Implantation of HCT116 or SW48 human CRC cells below the skin of immunodeficient mice prior to their orthotopic injection into the caecum generates models with enhanced metastatic efficiency in the lymph nodes, lung, liver and peritoneum, they report. Most notably, in the HCT116 model, subcutaneous preconditioning generates visible liver metastases that compromise survival. These findings suggest that *in vivo* models of CRC (and probably other cancers) generated using this new approach could facilitate the elucidation of the mechanisms of metastasis and the evaluation of anti-metastatic drugs. **Page 387**

